# A cell cycle–related lncRNA signature predicts the progression-free interval in papillary thyroid carcinoma

**DOI:** 10.3389/fendo.2023.1110987

**Published:** 2023-02-27

**Authors:** Shuang Li, Ming-Yu Ran, Hong Qiao

**Affiliations:** ^1^ Department of Endocrinology, The Second Affiliated Hospital of Harbin Medical University, Harbin, China; ^2^ College of Bioinformatics Science and Technology, Harbin Medical University, Harbin, China

**Keywords:** papillary thyroid carcinoma, cell cycle, long non-coding RNA, progression-free interval, prognostic signature

## Abstract

The cell cycle plays a vital role in tumorigenesis and progression. Long non-coding RNAs (lncRNAs) are key regulators of cell cycle processes. Therefore, understanding cell cycle–related lncRNAs (CCR-lncRNAs) is crucial for determining the prognosis of papillary thyroid carcinoma (PTC). RNA-seq and clinical data of PTC were acquired from The Cancer Genome Atlas, and CCR-lncRNAs were selected based on Pearson’s correlation coefficients. According to univariate Cox regression, least absolute shrinkage and selection operator (LASSO), and multivariate Cox regression analyses, a five-CCR-lncRNA signature (*FOXD2-AS1*, *LOC100507156*, *BSG-AS1*, *EGOT*, and *TMEM105*) was established to predict the progression-free interval (PFI) in PTC. Kaplan–Meier survival, time-dependent receiver operating characteristic curve, and multivariate Cox regression analyses proved that the signature had a reliable prognostic capability. A nomogram consisting of the risk signature and clinical characteristics was constructed that effectively predicted the PFI in PTC. Functional enrichment analyses indicted that the signature was involved in cell cycle– and immune-related pathways. Furthermore, we also analyzed the correlation between the signature and immune cell infiltration. Finally, we verified the differential expression of CCR-lncRNAs *in vitro* using quantitative real-time polymerase chain reaction. Overall, the newly developed prognostic risk signature based on five CCR-lncRNAs may become a marker for predicting the PFI in PTC.

## 1 Introduction

Thyroid cancer (TC) is the most common endocrine system malignancy and accounts for 3.4% of all cancers diagnosed globally each year (1), of which approximately 85% of TC cases are papillary thyroid carcinoma (PTC) (2). Although PTC has a good outcome, with a five-year survival rate of more than 97% (3), approximately 30% of patients exhibit recurrence and metastasis after conventional treatment (4) and have a poor prognosis. Satisfactory tools for accurately assessing prognosis are still lacking. Therefore, it is essential to develop a new reliable biomarker to accurately determine the prognosis of PTC patients.

Cell cycle dysregulation and genetic alterations in cell cycle–related regulatory proteins lead to the limitless proliferation and growth of tumor cells (5), and research has shown that various genes affect tumor cell progression by regulating the cell cycle. For instance, *PTBP3* promotes the proliferation of lung squamous cell carcinoma cells by *CDC25A*-mediated cell cycle progression (6), and *ZNF703* knockdown inhibits triple-negative breast cancer cell progression by inducing G1-phase arrest (7). Hence, targeting cell cycle control is a promising therapeutic strategy (8). In addition, the prognostic value of cell cycle–related genes (CCRGs) has been reported in certain tumors (9–11).

Long non-coding RNAs (lncRNAs) are defined as RNAs that are longer than 200 nucleotides and do not encode proteins (12). They primarily participate in epigenetic regulation, including chromatin modifications, splicing, and transcriptional and post-transcriptional regulation (13). In addition, lncRNAs are engaged in various cellular processes, including autophagy, differentiation, cell cycle regulation, proliferation, apoptosis, and mesenchymal stem cell differentiation (14). Many lncRNAs that are closely involved in the occurrence and progression of PTC have been reported, including lncRNA *H19* (15), *lnc-MPEG1-1* (16), and lncRNA *MIAT* (17). Furthermore, lncRNA signatures have been developed to determine prognosis in PTC (18–20). However, prognostic signatures based on cell cycle–related lncRNAs (CCR-lncRNAs) for PTC have not been developed.

In this study, we developed a cell cycle–related lncRNA signature (CCRLSig) for the prediction of the progression-free interval (PFI) in PTC using data from The Cancer Genome Atlas (TCGA). We validated the prognostic value of this CCRLSig and established a nomogram. Moreover, functional enrichment analysis and an analysis of immune cell infiltration were performed to explore the mechanism by which the signature contributes to prognosis. Finally, we used quantitative real-time polymerase chain reaction (qRT–PCR) to detect the differential expression of CCR-lncRNAs in PTC. Our results provide the first demonstration that a CCRLSig can effectively determine the prognosis of PTC and provide new perspectives for the development of therapeutic strategies.

## 2 Materials and methods

### 2.1 Data collection

HTseq-FPKM and HTseq-count data of TC samples were obtained from TCGA (https://cancergenome.nih.gov/, accessed January 24, 2022). After removing TC samples of other pathological types, a total of 502 PTC samples and 58 adjacent normal samples as well as the corresponding clinical information were obtained from TCGA.

### 2.2 Identification of CCR-lncRNAs

The CCRG sets were extracted from MsigDB (http://www.gsea-msigdb.org/gsea/index.jsp, accessed January 24, 2022). Differentially expressed genes (DEGs) between PTC and normal tissues were screened using DEseq2 (|log Fold Change (FC)| > 1, p.adjust< 0.05). Next, 266 differentially expressed CCRGs were identified from the intersection of CCRGs and DEGs. CCR-lncRNAs were acquired by Pearson correlation coefficient analysis between the 266 CCRGs and differentially expressed lncRNAs (DElncRNAs) in the PTC samples, and the Benjamini–Hochberg (BH) method was used for p-value correction (|*R*|> 0.4, BH< 0.05).

### 2.3 Construction and validation of a CCR-lncRNA prognostic signature

Post-operative recurrence and metastasis are the main factors leading to the poor prognosis of PTC; accordingly, the PFI was chosen as the endpoint of this study, instead of overall survival. A total of 498 PTC samples with complete PFI information were included after removing samples with incomplete clinical information and prognosis less than 30 days. The samples were categorized into training (249) and test (249) cohorts at a 1:1 ratio by the “caret” package. Univariate Cox regression analyses were primarily used to screen CCR-lncRNAs that were associated with PFI in the training cohort (p< 0.05). To reduce noise caused by gene interactions and co-expression patterns, we applied LASSO-Cox regression to filter lncRNAs; the formula is as follows:


 βmin∑i=1n(yi − ∑j=1qβjxij)2, subject to ∑j=1q|βj| ≤ π

where the β is the regression coefficient, x is the gene expression level, and π is an adjusted parameter decided by 10-fold cross validation. Parameter optimization was repeated 1000 times for all lncRNAs. These lncRNAs were utilized to construct a Cox proportional hazards model. The Akaike information criterion was adopted to select the appropriate model. The risk score for each patient (P_RS_) was calculated with the following formula:


$$\begin{array}{l} P_{RS}=[0\mathrm{.84919*normalized}\;\mathrm{expression}\;\mathrm{value}\;\mathrm{of}\;\mathrm{FOXD2-AS1]}\;-\;\mathrm{[3}\mathrm{.86735*}\\ \mathrm{normalized}\;\mathrm{expression}\;\mathrm{value}\;\mathrm{of}\mathrm{LOC100507156]}\;+\;\mathrm{[0}\mathrm{.67474*}\\ \mathrm{normalized}\;\mathrm{expression}\;\mathrm{value}\;\mathrm{of}\;\mathrm{BSG-AS1]+[0}\mathrm{.76288*}\\ \mathrm{normalized}\;\mathrm{expression}\;\mathrm{value}\;\mathrm{of}\;\mathrm{EGOT]+}\;\mathrm{[0}\mathrm{.46501*}\\ \mathrm{normalized}\;\mathrm{expression}\;\mathrm{value}\;\mathrm{of}\;\mathrm{TMEM105]} \end{array}$$

Thereafter, the cut-off value for the risk score was determined using the “survminer” package and patients were separated into high- and low-risk groups. The optimal cut-off values for the training, test and entire cohort were 2.026, 1.998 and 2.038, respectively. Kaplan–Meier (K–M) survival curves was plotted using the “survival” package. Time-dependent receiver operating characteristic (ROC) curves was generated using the “timeROC” package to assess the predictive accuracy of the model. In addition, clinicopathological differences between the high- and low-risk groups were analyzed using the Wilcoxon rank sum test.

### 2.4 Independent prognostic value determination and nomogram construction

To validate the CCRLSig as an independent prognostic indicator for PFI in PTC, the signature risk score and clinical parameters (age, gender, tumor size, T stage, N stage, AJCC stage, multifocality, aggressiveness, anatomic site of the tumor, and BRAFV600E mutation status) were subjected to univariate and multivariate Cox regression analyses.

A nomogram incorporating clinical and pathological factors and the risk score was established using the “survival” and “rms” R packages to predict 1-, 3- and 5-year PFI in the entire cohort. The calibration curves and ROC curves were used to estimate the predictive accuracy of the nomogram.

### 2.5 Functional enrichment analysis

DEGs between the high- and low-risk groups based on the CCRLSig were identified using DEseq2 with a |log FC| > 0.5 and p.adjust< 0.05 as thresholds, after which Gene Ontology (GO), Kyoto Encyclopedia of Genes and Genomes (KEGG) pathway, and gene set enrichment analysis (GSEA) of these DEGs were conducted using the R “clusterProfiler” package.

### 2.6 Tumor mutation burden (TMB) and immune cell infiltration

The somatic mutation data of PTC patients were acquired from TCGA. The TMB was compared between the high- and low-risk groups using the “maftools” package. The TMB was measured as follows: (total mutations/total covered bases) × 10^6 for each patient with PTC.

To further explore if the risk signature was associated with immune cell infiltration in PTC, immunity-associated data were extracted from xCell (http://xCell.ucsf.edu/) and parameters were compared between the high- and low-risk groups by the Wilcoxon rank sum test. In addition, correlations between the CCR-lncRNAs and immune cell fractions were evaluated.

### 2.7 Cell culture

The human normal thyroid cell line (Nthy-ori3–1) was provided by Professor Hongmei Shen (Center for Endemic Disease Control, Chinese Center for Disease Control and Prevention, Harbin Medical University, Harbin, China). The human PTC cell line (TPC-1) was obtained from the School of Public Health, Shandong First Medical University & Shandong Academy of Medical Sciences. The cell lines were certified by short tandem repeat (STR) validation. TPC-1 and Nthy-ori3–1 cells were cultured in RPMI 1640 supplemented with 10% fetal bovine serum (Moregate, Bulimba, Australia) and 1% penicillin/streptomycin solution (Beyotime Biotechnology, Shanghai, China) at 37°C and 5% CO_2_ conditions.

### 2.8 Quantitative real-time PCR validation

Total RNA was extracted from the cell lines using TRIzol reagent (Thermo Fisher Scientific, Waltham, MA, USA), and reverse transcribed into cDNA using the Roche Reverse Transcription Kit (Roche, Basel, Switzerland). QRT-PCR was performed using a real-time PCR instrument (Q5). Glyceraldehyde 3-phosphate dehydrogenase (*GAPDH*) served as a control. The primers for lncRNAs were provided by Sangon Biotech (Shanghai, China) and are listed in Table S1. The relative expression level of each lncRNA was calculated by the 2^-△△CT^ method.

### 2.9 Statistical analysis

The R statistical environment (V4.1.2) and GraphPad Prism 8.0 were used for statistical analyses. Analysis of variance, Wilcoxon rank sum test, and Student’s t-tests were applied to evaluate differences between groups. The K–M analysis and log-rank test were adopted for comparing PFIs between groups. Values of p< 0.05 were considered statistically significant difference for all analyses.

## 3 Results

### 3.1 Identification of CCR-lncRNAs

We identified 655 DElncRNAs and 266 CCRGs in a comparison between 502 PTC and 58 adjacent normal samples (|log FC| > 1, p.adjust< 0.05). Based on Pearson correlation coefficients between the CCRGs and lncRNAs (|R| > 0.4, p< 0.05), we obtained 446 CCR-lncRNAs.

### 3.2 Construction of a CCRLSig predicting PFI in PTC

**Figure 1:**
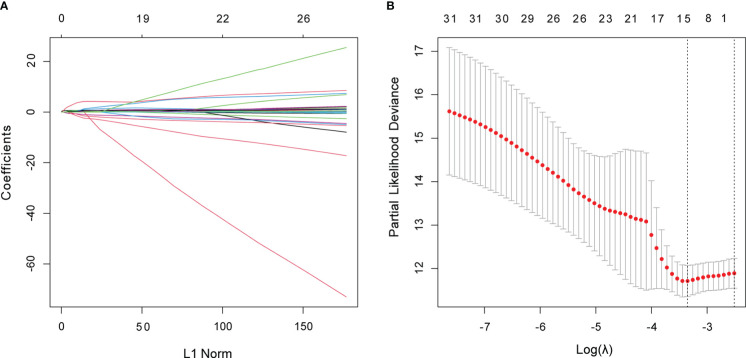
Identification of CCR-lncRNAs associated with PFI of PTC. **(A, B)** LASSO coefficient profiles of the PFI-associated CCR-lncRNAs. CCR-lncRNAs, cell cycle–related long non-coding RNAs; PFI, progression-free interval; PTC, papillary thyroid carcinoma; LASSO, least absolute shrinkage and selection operator.

**Figure 2:**
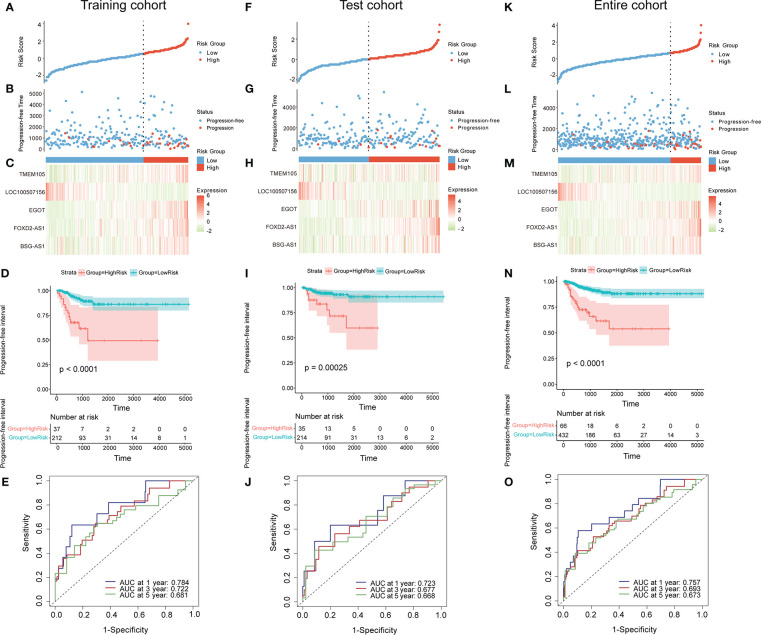
Construction and validation of a CCR-lncRNA-based prognostic signature. **(A, F, K)** PTC patients were sorted by risk score in the training, test, and entire cohort **(B, G, L)** PFI status of PTC patients in the training, test, and entire cohort. **(C, H, M)** Heatmap of the expression of five CCR-lncRNAs in the training, test, and entire cohort. **(D, I, N)** Kaplan–Meier curve analysis of the high- and low-risk groups in the training, test, and entire cohort. **(E, J, O)** Time-dependent ROC curves for 1-, 3-, and 5-year PFI predictions for the signature in the training, test, and entire cohort. CCR-lncRNAs, cell cycle–related long non-coding RNAs; PTC, papillary thyroid carcinoma; PFI, progression-free interval.

The 498 PTC samples with complete clinical information were divided into training and test cohorts at a ratio of 1:1. In the training cohort, 31 CCR-lncRNAs associated with PFI were selected by univariate Cox analyses. Subsequently, we applied LASSO-Cox regression analysis and established a prognostic signature consisting of five CCR-lncRNAs (*FOXD2-AS1*, *LOC100507156*, *BSG-AS1*, *EGOT*, and *TMEM105*; **Figures 1A, B**). The K–M survival curves for the five CCR-lncRNAs relating to the PFI are shown in **Supplementary Figures S1A–E**. Next, the risk score for each patient in the training cohort was calculated using the following formula: risk score = [(0.84919) × normalized expression value of *FOXD2-AS1*] – [(3.86735) × normalized expression value of *LOC100507156*] – [(0.67474) × normalized expression value of *BSG-AS1*] – [(0.76288) × normalized expression value of *EGOT*] – [(0.46501) × normalized expression value of *TMEM105*]. Next, according to the optimal cut-off value of the risk score, patients were grouped into high- and low-risk groups. As the risk score increased, patients’ prognosis worsened (**Figures 2A, B**). The expression levels of *TMEM105*, *EGOT*, *FOXD2-AS1*, and *BSG-AS1* in the CCRLSig increased and the expression level of *LOC100507156* decreased as the risk score increased (**Figure 2C**). K–M analysis showed that PTC patients in the high-risk group had a shorter PFI than that of patients in the low-risk group (**Figure 2D**). The area under the ROC curve (AUC) values for the risk score for 1-, 3-, and 5-year PFI were 0.784, 0.722, and 0.681, respectively (**Figure 2E**).

### 3.3 Validation of the CCRLSig

To validate the accuracy of the signature, patients in the test cohort and the entire cohort were separated into high- and low-risk groups according to the optimal cut-off value for each dataset. The risk curves, PFI status, and heatmaps of risk lncRNA expression profiles in the test cohort and entire cohort were consistent with those of the training cohort (**Figures 2F–H, K–M**). Similarly, a K–M curve analysis of the test cohort and entire cohort indicated that the high-risk groups had a shorter PFI than the low-risk groups (**Figures 2I, N**). In the test cohort, the AUC values for the risk score for 1-, 3-, and 5-year PFI were 0.723, 0.677, and 0.668, separately (**Figure 2J**). In the entire cohort, the AUC values for the risk score for 1-, 3-, and 5-year PFI were 0.757, 0.693, and 0.673, separately (**Figure 2O**). These results suggest that the five-CCR-lncRNA risk signature has good predictive performance for PFI in PTC.

### 3.4 Independent prognostic value of the CCRLSig

**Figure 3:**
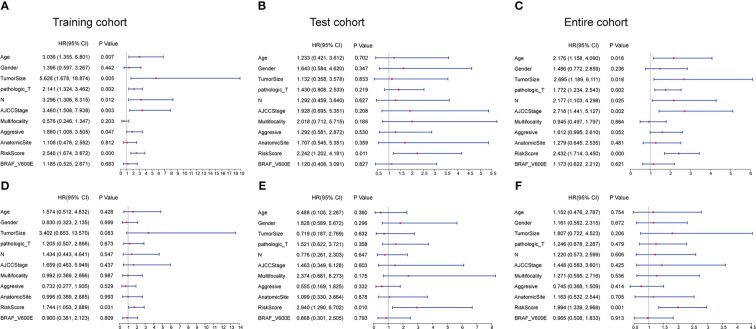
Univariate and multivariate Cox regression analyses of the signature and clinical characteristics. **(A–C)** Univariate Cox regression analysis of the signature risk score and clinical characteristics in the training, test, and entire cohort. **(D–F)** Multivariate Cox regression analysis of the signature risk score and clinical characteristics in the training, test, and entire cohort.

Univariate and multivariate Cox regression analyses were employed to explore whether the risk score based on the CCRLSig predicts PFI in PTC. Univariate Cox analyses showed that the risk score was notably correlated to the PFI in the training, test, and entire cohorts (**Figures 3A–C**). A multivariate Cox analysis proved that the risk score was an independent predictor for the PFI of PTC in the training, test, and entire cohorts (**Figures 3D–F**).

### 3.5 Correlations between the CCRLSig and clinicopathological characteristics

**Figure 4:**
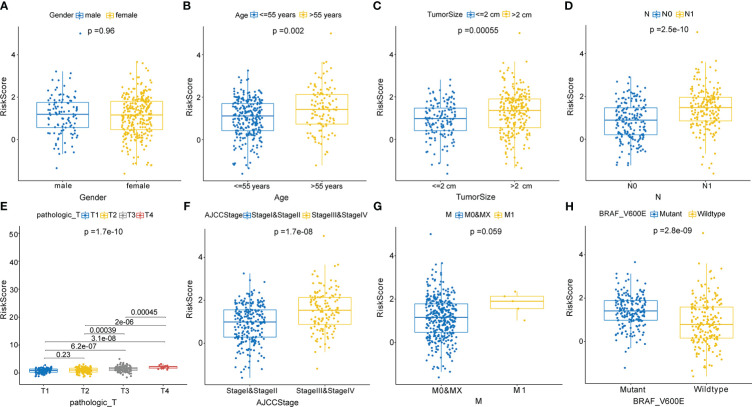
Correlations between the risk score based on the signature and clinical characteristics. **(A)** Male and female. **(B)** Age ≤ 55 years and > 55 years. **(C)** Tumor size ≤2 cm and > 2 cm. **(D)** N0 and N1 stage. **(E)** T1, T2, T3, and T4 stage. **(F)** AJCC stages I and II and stages III and V. **(G)** M0, MX, and M1stage. **(H)** Mutant and wild-type BRAFV600E.

In correlation analyses, the risk scores based on the CCRLSig were higher for patients older than 55 years than for patients younger than 55 years (**Figure 4B**). The risk scores of tumor size >2 cm were higher than those for tumor size ≤ 2 (**Figure 4C**). The risk scores for N1 were higher than those for N0 (**Figure 4D**). We also found that the risk score tended to increase with T stage and AJCC stage (**Figures 4E, F**), implying the critical role of the signature in the progression of PTC. Additionally, the risk score was significantly higher in patients with the *BRAFV600E* mutation than in those without the mutation (**Figure 4H**). Nevertheless, there were no significant correlations between the risk score and gender or M stage (p > 0.05, **Figures 4A, G**).

### 3.6 Construction and assessment of a nomogram

**Figure 5:**
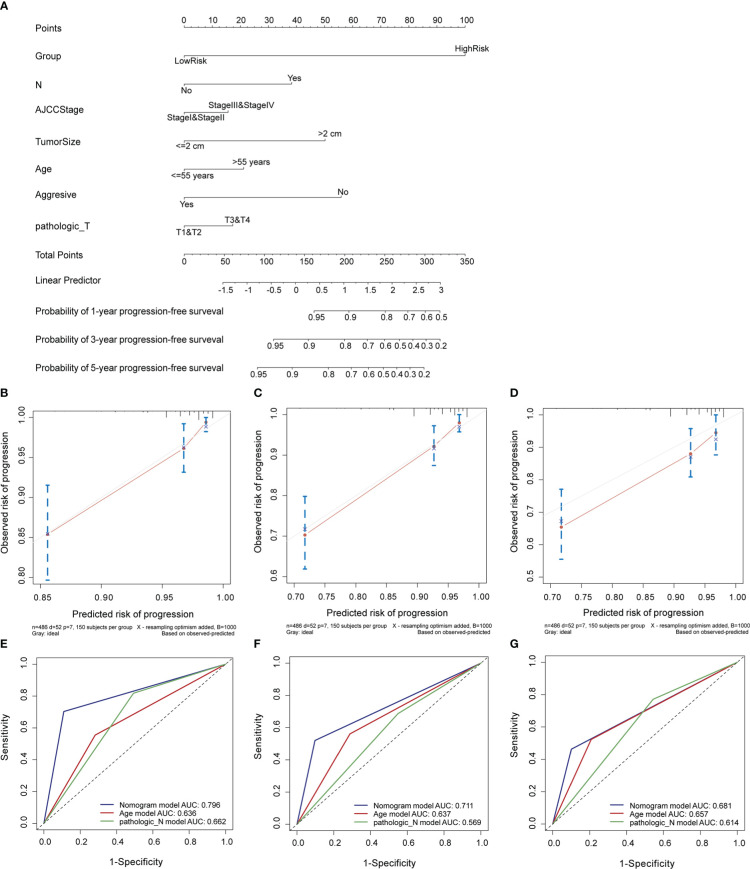
Construction and evaluation of a nomogram in the entire cohort. **(A)** Nomogram established to predict 1-, 3-, and 5-year PFI of PTC. **(B–D)** Calibration curves assessed the concordance between predicted and observed 1-, 3-, and 5-year PFI. **(E–G)** The ROC curves of the nomogram and other clinical characteristics at 1-, 3-, and 5-year time points. PFI, progression-free interval.

In the entire cohort, we built a nomogram for the prediction of the 1-, 3-, and 5-year PFI of PTC based on the signature’s risk score and significant clinicopathological features (P< 0.05) identified in univariate Cox regression analyses, including the pathological T stage, N stage, AJCC stage, tumor size, age, and aggressiveness (**Figure 5A**). The calibration curve indicated that the nomogram-predicted PFI at one, three, and five years was highly consistent with the practically observed PFI (**Figures 5B–D**). Furthermore, the AUCs of the nomogram for evaluation of 1-, 3-, and 5-year PFI were 0.796, 0.711, and 0.681, respectively, and the predictive performances were superior to those of other clinical characteristics (age and N stage; **Figures 5E–G**). These results suggest that the nomogram reliably predicts the 1-, 3-, and 5-year PFI in PTC.

### 3.7 Functional enrichment analysis

**Figure 6:**
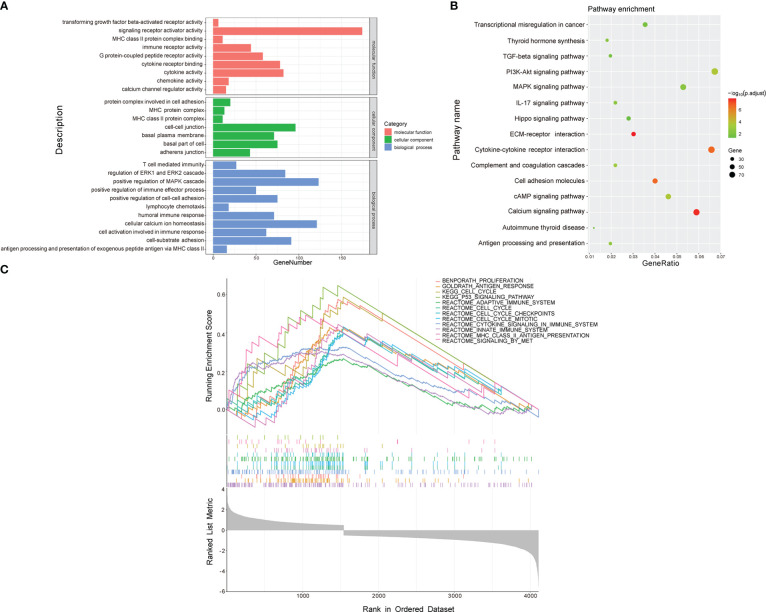
Functional enrichment analysis of DEGs between low- and high-risk groups. **(A)** GO analysis. **(B)** KEGG pathway analysis. **(C)** GSEA.

To investigate the molecular mechanisms and pathways by which the signature is related to the risk of PTC progression, we carried out GO and KEGG enrichment analyses and GSEA of DEGs between the two risk groups. The GO enrichment analysis demonstrated that the DEGs were enriched in multiple biological processes and molecular functions, including cellular calcium ion homeostasis, positive regulation of MAPK cascade, cell-substrate adhesion, positive regulation of cell-cell adhesion, humoral immune response, T cell mediated immunity, and chemokine activity (**Figure 6A**). The KEGG analysis showed that the DEGs were involved in the PI3K-Akt, MAPK signaling pathway, cytokine-cytokine receptor interaction, cell adhesion molecules, antigen processing and presentation, and IL-17 signaling pathway (**Figure 6B**). The GSEA revealed that the DEGs were enriched in the cell cycle pathway and several immune-related biological processes (**Figure 6C**), including the cell cycle, P53 signaling pathway, cell cycle checkpoints, innate immune system, antigen response, and MHC class II antigen presentation.

### 3.8 Analysis of TMB

To reveal genetic variation in risk score subtypes, we compared the TMB between the high- and low-risk groups. Compared with the low-risk group, the high-risk group had a markedly higher TMB (p< 0.01, **Supplementary Figure S2A**). The top 20 mutated genes in two risk group are shown in **Supplementary Figures S2B, C**. We observed that the mutation rate of *BRAF* was markedly higher in the high-risk group (84%) than in the low-risk group (56%). **Supplementary Figures S2D, E** show a complete view of the somatic mutations in the high- and low-risk groups.

### 3.9 Relationship between the signature and immune cell infiltration

**Figure 7:**
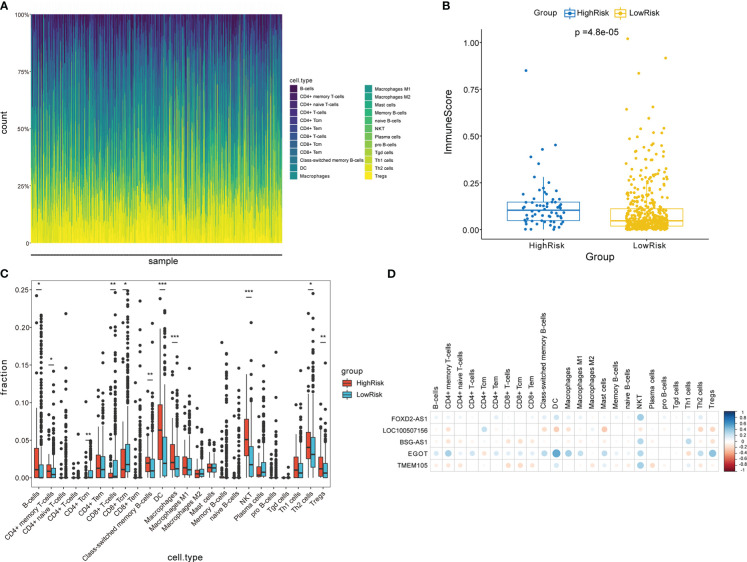
Analysis of immune cell infiltration in two risk groups stratified by the signature. **(A)** Overview of immune cell infiltration in each patient with PTC in the entire cohort. **(B)** Comparison of immune scores between two risk groups. **(C)** Analysis of immune cell infiltration in two risk groups. **(D)** Correlation analyses between five CCR-lncRNAs and immune cell infiltration. PTC, papillary thyroid carcinoma; CCR-lncRNAs, cell cycle–related long non-coding RNAs. (*P< 0.05, **P< 0.01, ***P< 0.001).

The functional enrichment analysis displayed that the CCRLSig may be associated with immunity. Hence, we further analyzed the relationships between the signature and immune cell infiltration. The relative frequencies of infiltrating immune cells in all PTC patients are shown in **Figure 7A**. The high-risk group exhibited higher immune scores than the low-risk group (**Figure 7B**). The fractions of B-cells, CD4+ memory T cells, class-switched memory B-cells, DC, macrophages, NKT, Th2 cells, and Tregs in the high-risk group were markedly higher than those in the low-risk group (p< 0.05). In contrast, the fractions of CD4+ Tcm, CD8+ T cells, and CD8+ Tcm cells in the high-risk group were lower than those in the low-risk group (**Figure 7C**). Additionally, we explored correlations between the expression levels of the five lncRNAs in the signature and the infiltration of multiple immune cells in PTC (**Figure 7D**). These results implied that the signature is linked to immune cell infiltration and may regulate immune processes in PTC.

### 3.10 Validation of the expression levels of five CCR-lncRNAs in cell lines

**Figure 8:**
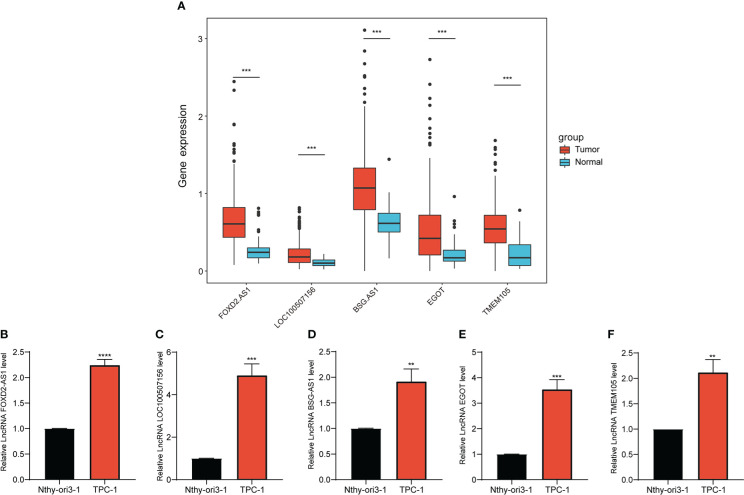
Expression of selected CCR-lncRNAs in cell lines. **(A)** Differential expression of five CCR-lncRNAs between normal tissues and tumor tissues of PTC of TCGA. **(B–F)** Relative expression of five CCR-lncRNAs in a PTC cell line and normal human thyroid cell line. CCR-lncRNAs, cell cycle–related long non-coding RNAs; PTC, papillary thyroid carcinoma (*P< 0.05, **P< 0.01, ***P< 0.001).

We analyzed the differential expression of the five lncRNAs between normal and PTC tissues in TCGA data, as illustrated in **Figure 8A**, and thereafter verified the results in cell lines. The expression levels of *FOXD2-AS1*, *LOC100507156*, *BSG-AS1*, *EGOT*, and *TMEM105* were notably higher in TPC-1 cells than in Nthy-ori3–1 cells (**Figures 8B–F**), which was in line with the results of the bioinformatics analysis, thereby supporting the accuracy of our analysis.

## 4 Discussion

The cell cycle is closely related to the growth and proliferation of cancer cells (21), and numerous lncRNAs related to the progression of cancers *via* cell cycle regulation have been identified (22). Understanding the expression levels of these lncRNAs and their combined regulatory patterns is crucial for determining patient outcomes and prognosis. Therefore, we constructed a cell cycle–related lncRNA signature and explored its prognostic capability in PTC patients.

We screened out CCR-lncRNAs and divided PTC samples into training and test cohorts to establish and validate a prognostic signature. Univariate Cox and LASSO-Cox regression analyses were employed to construct a CCRLSig for predicting the PFI of PTC in the training cohort. The prognostic value of the CCRLSig was supported by K–M curve analysis, ROC curve analyses, and multivariate Cox analysis. Furthermore, a nomogram illustrated that the signature has excellent predictive power. To further understand the clinical application of the CCRLSig, we investigated its association with clinicopathological characteristics and observed that a high risk score was positively correlated with age, tumor size, BRAFV600E mutation, AJCC stage, N stage, and T stage. These results indicated that the CCRLSig effectively predicts outcome and can better guide risk stratification for PTC management.

To better understand the underlying mechanisms by which this CCRLSig affects the prognosis of PTC, we performed a functional enrichment analysis of DEGs between the two risk groups. GO and KEGG analyses indicated that these DEGs were enriched in the following terms and pathways: cell-substrate adhesion, cell adhesion molecules, PI3K-Akt signaling pathway, MAPK signaling pathway, humoral immune response, T cell mediated immunity, and the IL-17 signaling pathway, all of which are associated with tumor proliferation, migration and immunity. GSEA also demonstrated that these DEGs were primarily engaged in cell cycle– and immune-related signaling pathways. These results imply that CCR-lncRNAs can affect the progression of PTC by regulating cell cycle– and immune-related signaling pathways, providing new directions for the treatment of PTC.

Recent studies had indicated the key roles of lncRNAs in the regulation of cancer immunity, including lncRNAs involved in immune cell differentiation, proliferation, trafficking, and infiltration (23). For example, the lncRNA *HOXA-AS2* promotes Treg proliferation and immune tolerance in glioma (24), and *LINC00887* promotes clear cell renal cell carcinoma progression by inhibiting the infiltration of CD8+ T cells (25). CCRG signatures are potential indicators of immune cell infiltration, immune evasion, and immune responses (26–28). Our previous functional enrichment analysis has shown that the CCRLSig was involved in immune processes. Thus, we further analyzed immune cell infiltration in the two risk groups. The high-risk group had infiltrates with higher proportions of B-cells, CD4+ memory T cell, DC, macrophages, NKT, Th2, and Tregs than the low-risk group, and the low-risk group primarily showed the infiltration of CD8+T cells, CD4+ Tcm, and CD8+Tcm cells. Studies have shown that Tregs and DCs play crucial roles in tumor immune escape (29–31) and promote tumor progression. However, CD8+ T cells exert an antitumor effect in PTC (32). Our results demonstrated that low-risk patients had a lower risk of immune evasion and may be more responsive to immunotherapy. Additionally, in PTC, DCs are significantly related to tumor T stage (T3/T4) and lymph node metastasis (33). Tregs show elevated infiltration in the thyroid tissue of PTC patients and were positively correlated with an advanced disease stage (34). CD8+ T cell infiltration is correlated with a lower incidence of lymph node metastasis and favorable prognosis in TC (35, 36). These findings further suggest that the low-risk group has a better prognosis. In summary, our findings suggested that the CCRLSig is associated with tumor immunity and can predict the immune landscape in PTC patients. In addition, these CCR-lncRNAs may be targets for immunotherapy.

Most of the lncRNAs in our signature have been previously reported to be implicated in cancers. For example, lncRNA *FOXD2-AS1* has been discovered to be upregulated in PTC and correlated with a poor prognosis (37), consistent with our results. In addition, lncRNA *FOXD2-AS1* promotes the progression of multiple cancers by participating in several biological processes, such as chemo-resistance, proliferation, migration and invasion (38–40). The lncRNA *EGOT* may play different roles in different types of cancers. It promotes the progression of hepatocellular carcinoma (41), colon cancer (42), and gastric cancer (43). However, another study has shown that the lncRNA *EGOT* inhibits the progression of breast carcinoma (44) and renal cell carcinoma (45). The lncRNA *TMEM105*, a ferroptosis and immune-related lncRNA, serves as prognostic and diagnostic biomarker for patients with breast-infiltrating duct and lobular carcinoma (46). The lncRNA *BSG-AS1* contributes to the proliferation and metastasis of hepatocellular carcinoma *via* maintaining *BSG* mRNA stability (47). However, *LOC100507156* has not yet been reported in cancer and requires further investigations. These previous findings indicate that CCR-lncRNAs participate in the progression of multiple types of tumors, further indicating that it is reasonable to develop a risk signature based on CCR-lncRNAs to determine prognosis in PTC. In addition, the expression differences of the five CCR-lncRNAs were verified at the cellular level.

Although the newly constructed CCRLSig may be applied to predict the outcome of PTC, our study had some deficiencies. First, the dataset used to construct and validate the prognostic signature based on CCR-lncRNAs was obtained only from TCGA. Additional external data from other public databases are needed to evaluate the reliability of the signature. Second, we conducted a preliminary expression study of five CCR-lncRNAs in the signature at the cellular level. However, further functional analyses and mechanistic studies are needed. We will conduct more in-depth studies to verify the performance of our CCRLSig.

In summary, we developed a new CCRLSig that can reliably predict the PFI of PTC, providing a new direction for the prognostic management and treatment of PTC.

## Data availability statement

The datasets presented in this study can be found in online repositories. The names of the repository/repositories and accession number(s) can be found in the article/**Supplementary Material**.

## Author contributions

SL and HQ conceived and designed the study, SL wrote the manuscript, SL and MR collected the data and performed bioinformatics analysis. All authors contributed to the article and approved the submitted version
