# Supplementary material for: A cell cycle–related lncRNA signature predicts the progression-free interval in papillary thyroid carcinoma
Source: Front Endocrinol (Lausanne). 2023 Feb 27;14:1110987. doi: 10.3389/fendo.2023.1110987 (PMC10009218; doi:10.3389/fendo.2023.1110987)
Supplement: Supplementary file 1 [file DataSheet_1.docx]

A Cell Cycle-Related lncRNA Signature Predicts the Progression-Free Interval in Papillary Thyroid Carcinoma

Supplementary Material

Shuang Li^1^, Mingyu Ran^2^, Hong Qiao^1*^

*** Correspondence:** Hong Qiao* [qiaohong@hrbmu.edu](mailto:qiaohong@hrbmu.edu).cn

# Supplementary Figures and Tables

## Supplementary Table 1. The primer sequences of five cell ****cycle–related lncRNAs****.

| **Primer Name** | **Sequence (5'→3')** |
| --- | --- |
| TMEM105-f | AGTGCAGTGGTGCGATCTTG |
| TMEM105-r | TGCACTTCTGTAGTCCCAGCTACT |
| FOXD2-AS1-f | ACCTGTACCCACGCTTCAAAA |
| FOXD2-AS1-r | GCAGTGTGGCCTGAGAATGA |
| EGOT-f | TGCACAGGGAAACACAAATCA |
| EGOT-r | GCACGTGCTCCTTTTTAACCA |
| BSG-AS1-f | ACGCGTAAACCCAGACTCTCA |
| BSG-AS1-r | TCTTGCTGGCTGGTCCACTT |
| LOC100507156-f | GCAAAGCTGCCAAAGTGACA |
| LOC100507156-r | CTGGTCGACTTCCAGGTCCTT |
| GAPDH-f | GAAGGTCGGAGTCAACGGATT |
| GAPDH-r | CCTGGAAGATGGTGATGGGATT |

## Supplementary Figures


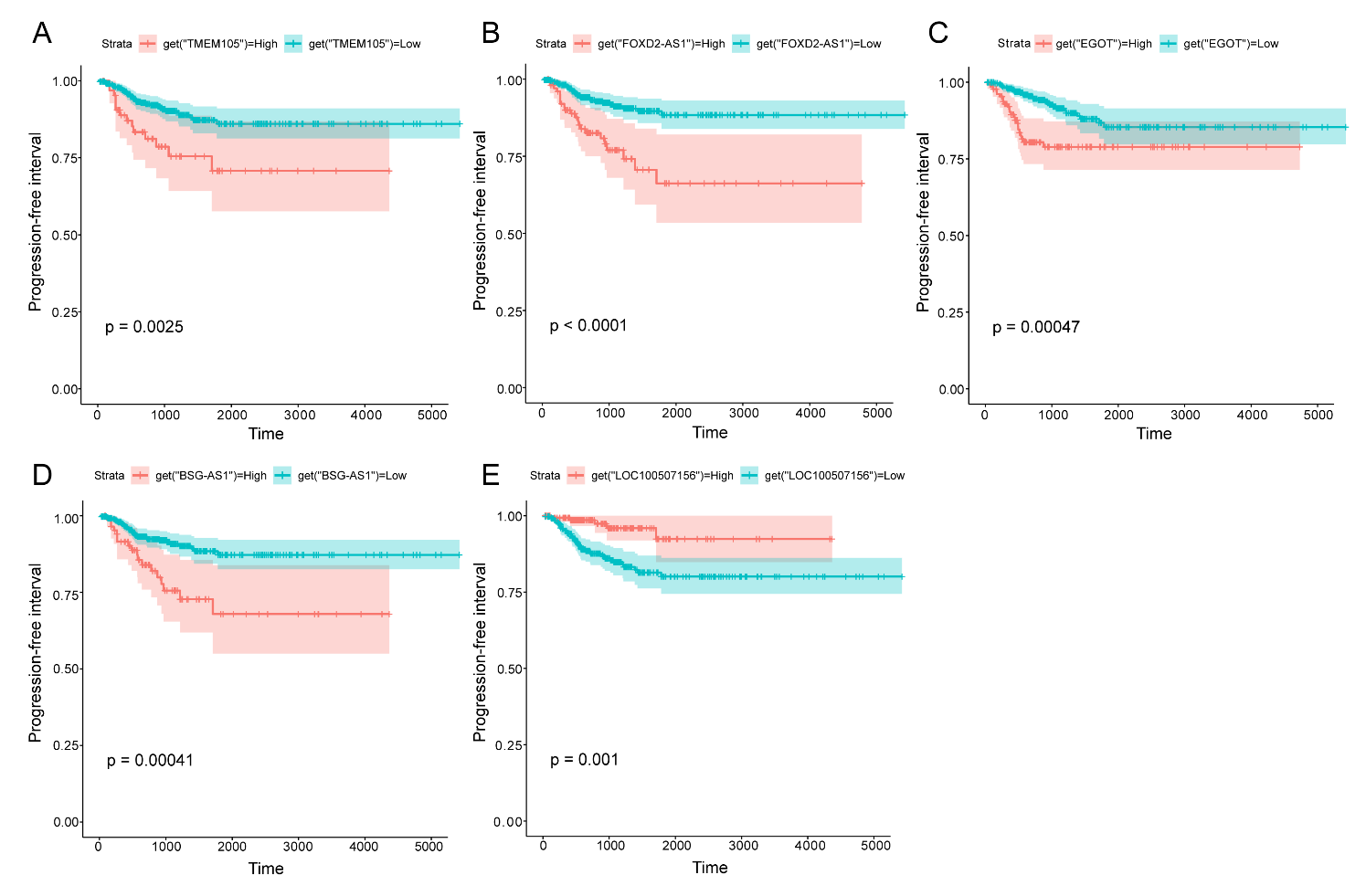


**Supplementary Figure 1. Kaplan–Meier survival curve analysis of five cell cycle–related lncRNAs. (A)** TMEM105, **(B)** FOXD2-AS1, **(C)** EGOT, **(D)** BSG-AS1, **(E)** LOC100507156.


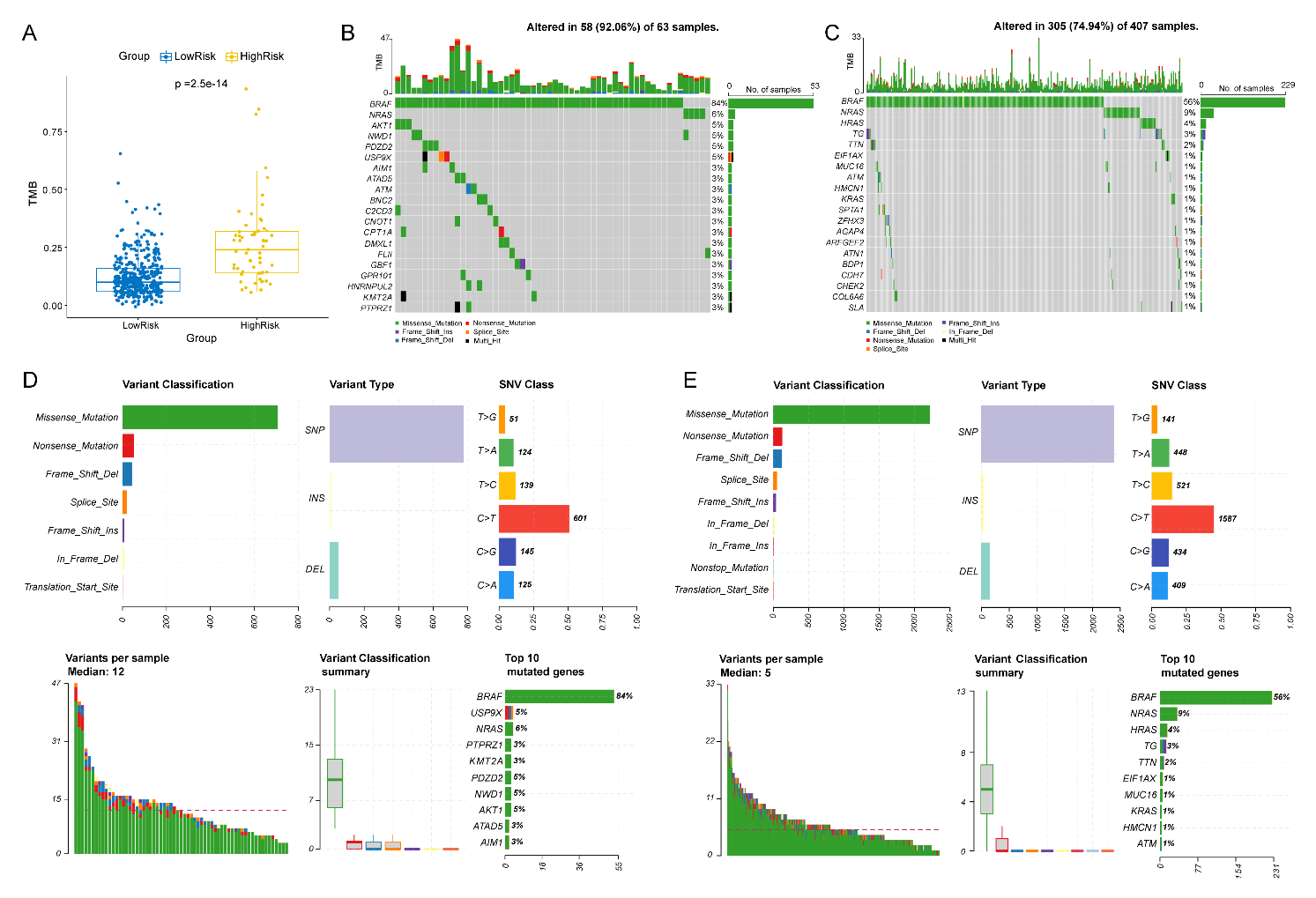


**Supplementary Figure 2. Analysis of the TMB in high- and low-risk groups.** (A) Comparison of the TMB between the high- and low-risk group, (B, C) Waterfall plot displays information on the top 20 mutated genes in the high- and low-risk group, (D, E) Entire view of somatic mutations in the high- and low-risk group. TMB, tumor mutation burden.
